# The role of kinases in peripheral nerve regeneration: mechanisms and implications

**DOI:** 10.3389/fneur.2024.1340845

**Published:** 2024-04-16

**Authors:** Xu Zhang, Xuchu Duan, Xiaoyu Liu

**Affiliations:** ^1^Key Laboratory of Neuroregeneration of Jiangsu and Ministry of Education, Co-innovation Center of Neuroregeneration, School of Life Science, Nantong Laboratory of Development and Diseases, Medical College, Clinical Medical Research Center, Affiliated Wuxi Clinical College of Nantong University, Nantong University, Nantong, China; ^2^Clinical Medical Research Center, Wuxi No. 2 People's Hospital, Jiangnan University Medical Center, Wuxi, China

**Keywords:** peripheral nerve regeneration, peripheral nerve injury, kinase, molecular mechanism, microenvironment

## Abstract

Peripheral nerve injury disease is a prevalent traumatic condition in current medical practice. Despite the present treatment approaches, encompassing surgical sutures, autologous nerve or allograft nerve transplantation, tissue engineering techniques, and others, an effective clinical treatment method still needs to be discovered. Exploring novel treatment methods to improve peripheral nerve regeneration requires more effort in investigating the cellular and molecular mechanisms involved. Many factors are associated with the regeneration of injured peripheral nerves, including the cross-sectional area of the injured nerve, the length of the nerve gap defect, and various cellular and molecular factors such as Schwann cells, inflammation factors, kinases, and growth factors. As crucial mediators of cellular communication, kinases exert regulatory control over numerous signaling cascades, thereby participating in various vital biological processes, including peripheral nerve regeneration after nerve injury. In this review, we examined diverse kinase classifications, distinct nerve injury types, and the intricate mechanisms involved in peripheral nerve regeneration. Then we stressed the significance of kinases in regulating autophagy, inflammatory response, apoptosis, cell cycle, oxidative processes, and other aspects in establishing conductive microenvironments for nerve tissue regeneration. Finally, we briefly discussed the functional roles of kinases in different types of cells involved in peripheral nerve regeneration.

## 1 Introduction

Peripheral nerves are defined as nerves that are not part of the brain or spinal cord but instead connect the central nervous system (CNS) to target organs for neural signal transduction. Peripheral nerve injury (PNI) is a prevalent disease condition resulting from either physical injury, including traumatic events and surgical procedures, or other disease conditions, such as diabetes, and autoimmune diseases like Guillain-Barre syndrome, systemic lupus erythematosus, and rheumatoid arthritis (1, 2). Iatrogenic injury, primarily caused by traction, cutting, surgery, and neuroma, is a common way to disrupt the continuity of axons and leads to sensory and motor dysfunction in the innervated region, significantly compromising patients' quality of life (3–5). In certain regions, the incidence of PNI caused by traffic accidents or mechanical injuries within the body has steadily risen alongside economic development (6).

There are different treatment therapies for disease condition-induced PNI, which depends on the causal diseases. For example, glucose control is the predominant method to prevent diabetic neuropathy, along with medicines for pain management (7). Guillain-Barre Syndrome (GBS), an autoimmune disorder involving demyelination of peripheral nerves, is the most common cause of acute flaccid paralysis worldwide. Traditional treatments for GBS encompass corticosteroids, plasma exchange, and intravenous (IV) administration of immunoglobulins (IVIG). Several novel therapies, such as complement inhibitors and cerebrospinal fluid (CSF) filtration, have been developing recently (8). Some patients with systemic lupus erythematosus can also get peripheral neuropathy, who are typically treated with corticosteroids and immunosuppression (9). For Sjogren's syndrome, another common autoimmune disease, apart from the clinical usage of gabapentin and pregabalin for pain relief, immunomodulatory and immunosuppressive therapies have been tested in trials (10).

Unlike the CNS, peripheral nerves possess an inherent capacity for self-repair and regeneration following injury. The treatment methods for physical PNI have evolved from initial microsurgery to current approaches, including autologous/allogeneic nerve tissue transplantation and tissue engineering material transplantation (11). However, the efficacy of these methods is exceptionally constrained due to the intricate nature of peripheral nerve differentiation and the limited understanding of the regeneration mechanisms involved in injured peripheral nerves. Consequently, it is imperative to identify and elucidate additional determinants and mechanisms that influence the regeneration process of the peripheral nerve system (PNS).

To date, numerous factors and molecules, such as Schwann cells (SCs), inflammation factors, kinases, and growth factors, have been identified as crucial regulators of peripheral nerve regeneration (PNR) (11–13). Kinases, a type of biochemical molecules ubiquitously present in cellular organisms, can catalyze the transfer of high-energy phosphate groups from high-energy donors (e.g., ATP) to substrates, primarily facilitating the transduction of various biological signals within cells. Due to their pivotal involvement in signal transduction, malfunctioning kinases often have severe detrimental effects and are associated with a series of diseases (14, 15). Here, we will review the recent progress of research on the mechanisms of PNR from the perspective of kinases.

## 2 Classification of kinases

The currently recognized kinases encompass protein kinases (including serine/threonine kinases and tyrosine kinases), lipid kinases, glucokinase (such as hexokinase and fructokinase), and others, with protein kinases comprising the majority. Over 500 protein kinases have been identified in the human body, encoded by more than 900 genes, accounting for ~5% of the human genome (16, 17).

### 2.1 Protein kinase

Based on the presence of phosphorylated groups on the receptors, protein kinases are categorized into various types, such as serine/threonine kinases, tyrosine kinases, histidine/lysine/arginine kinase, and aspartate/glutamate kinase, among others (18–24). Furthermore, protein kinases can also be classified based on their roles in signaling pathways, including AGC (Protein kinase PKA, PKG, PKC), calmodulin kinases (CaMK), mitogen-activated protein kinase (MAPK), MAPKK kinase (Raf), protein kinase B (AKT), Cyclin-dependent kinases (CDKs), protein tyrosine kinases (PTK), and other kinase families (25–31). Protein kinases play significant roles in diverse biological processes by activating target proteins and regulating cellular signaling transduction. Mutations and dysfunctions in protein kinases have been closely linked to human diseases, particularly cancer and inflammation. Consequently, protein kinases have emerged as promising pharmaceutical targets in medical applications (32, 33).

#### 2.1.1 Serine/threonine kinase and tyrosine kinase

The two primary types of protein kinases are serine/threonine kinases (STK) and tyrosine kinases (TK), which were among the earliest kinases to be identified (34). STKs catalyze the phosphorylation of serine/threonine hydroxyl groups on target proteins, while TKs facilitate the phosphorylation of tyrosine residues. These kinases are ubiquitously found in nearly all eukaryotic multicellular organisms and are crucial in mediating cellular signal transduction (35, 36). Based on the localization, these kinases can be categorized into transmembrane receptor kinases and cytoplasmic kinases. Functionally, the transmembrane kinases bind with extracellular ligands to transmit signals into the cells. In contrast, the cytoplasmic kinases are indispensable for transmitting intercellular signals, which subsequently regulate a wide range of cellular processes encompassing cell growth, metabolism, differentiation, proliferation, division, and apoptosis through the modulation of the gene expressions in the nucleus (37, 38).

Various STKs have been identified, including PKA, PKC, CaMK, pyruvate dehydrogenase kinase (PDK), and DNA-dependent protein kinase (DNA-PK). PKA, a typical protein kinase, is widely recognized for its crucial involvement in regulating various biological processes through the cAMP/PKA signaling pathway (39). TKs, based on their location and function, can be broadly categorized into receptor tyrosine kinases (RTKs), which are transmembrane receptor proteins, and non-receptor tyrosine kinases (NRTKs), which are found in the cytoplasm and nucleus. RTKs can be further classified into two groups, which are tyrosine kinase receptor (TKR) and tyrosine kinase associated receptor (TKAR) (40). Non-receptor kinases include PTK, TEC, and Janus kinase (JAK) family members (35, 41). Numerous tyrosine kinases identified thus far have been found to originate from proto-oncogenes. For instance, the initial two categories of NRTKs were identified as resulting from structural alterations of typical tyrosine kinases (35). Furthermore, these kinases also play a role in regulating inflammation and immune processes, as evidenced by the rapid tyrosine phosphorylation of diverse proteins during the proliferation of both standard and malignant tumor cells, as well as the activation of T cells, B cells, and mast cells (42, 43).

#### 2.1.2 Histidine/lysine/arginine kinase

Histidine protein kinases (HPK) are signaling enzymes that can phosphorylate conserved histidine residues. The phosphorylation of arginine and lysine residues follows processes similar to histidine due to their analogous basic groups. HPKs and their downstream target proteins form a two-component signal transduction system (44). A typical HPK is a transmembrane receptor containing an extracellular receptor region at the N-terminal end and an intracellular signal region at the C-terminal end. Despite the low similarity between HPK, STK, and TK, reports have suggested a potentially distant evolutionary relationship among them (45).

#### 2.1.3 Cysteine-rich receptor-like kinase

A receptor-like kinase (RLK) is a widely present protein kinase in plants, playing a crucial role in regulating various biological processes, including development, stress adaptation, and plant defense (46). A cysteine-rich receptor-like kinase (CRK), DUF26 receptor kinase, is pivotal in numerous signaling pathways. Functioning as a plasma membrane receptor, it recognizes and receives external signals and mediates subsequent intracellular signal transmission via phosphorylation (47).

#### 2.1.4 Aspartate/glutamate kinase

Aspartate kinase is the initial pivotal enzyme in the biosynthetic pathway of aspartate amino acids. It is widely distributed and exerts crucial roles in the metabolic pathways of plants and microorganisms. N-acetyl glutamate kinase, commonly known as NAGK, functions as the second rate-limiting enzyme in the biosynthesis of L-arginine, and it is also susceptible to feedback inhibition by the end product L-arginine (48).

### 2.2 Lipid kinase

Lipid kinases primarily encompass phosphoinositide lipid kinases (PIK), a class of proteins that facilitate the catalysis of phosphorylated variants of specific phosphatidylinositols. In mammals, the PIK family consists of three main categories: phosphatidylinositol 3 kinase (PI3K), phosphatidylinositol 4 kinase (PI4K), and phosphatidylinositol P (PIP) kinase (PIPK) (49). These PIK family members play crucial roles in signal transduction, generating second messengers that regulate cellular metabolism, promote overall wellbeing, and are indispensable for sustaining the energy necessary for cell growth and survival. Therefore, these lipid kinases are potential therapeutic targets for various diseases (50, 51).

### 2.3 Sugar kinase

Hexokinases and galactokinases are the most critical sugar kinases involved in sugar metabolism. Hexokinases typically phosphorylate glucose at the 6-position, while galactokinases catalyze the phosphorylation at the 1-position of galactose (52). Glucokinase (GK) is one of the isoenzymes of hexokinases. As a crucial glucose sensor in the human body, GK is primarily localized in pancreatic β cells and the liver, responsible for monitoring alterations in glucose concentration and activating the blood glucose regulation system to maintain blood glucose homeostasis (53).

## 3 Peripheral nerve injury

PNI is a prevalent affliction that primarily results in impaired nerve conduction in patients, ultimately leading to sensory and motor impairments and potentially lifelong disability (1). PNI can be mainly divided into mechanical injury and iatrogenic injury.

### 3.1 Mechanical injury

Mechanical injury pertains to nerve damage caused by external forces, such as traffic accidents, warfare, earthquakes, and industrial accidents. The injuries can be categorized into three types: crush injuries, transection injuries, and missing injuries. Crush injury is considered the least severe form of mechanical injury. In fundamental research, the sciatic nerve transection model is the most commonly utilized model for studying nerve damage (54). This model enables the investigation of various aspects, including the development, structure, and material transport within axons of peripheral nerves, such as chemical substances, signal molecules, and physical and chemical factors. Transection injury is a mechanical trauma that severs nerve tissue without inducing a defect. Transection injuries present greater challenges in terms of recovery compared to crush injuries, as they involve the breakage of nerve axons and endoneurium. Microsurgery, commonly employed as a treatment for transection, entails surgical sutures to restore the functionality of the injured nerve, typically improving functional recuperation (55). Missing injury, also known as nerve avulsion, is one of the most severe forms of mechanical injury, resulting in irreversible nerve damage. The treatment for missing injury depends on the gaps of the missing part. Autologous nerve transplantation or allogeneic nerve transplantation techniques are commonly employed in cases with small gaps. In contrast, larger gaps often require nerve conduits to facilitate regrowth and recovery (56–58).

### 3.2 Iatrogenic injury

Iatrogenic nerve lesions are nerve injuries that occur as a result of medical treatment or surgical procedures; for instance, the neurological dysfunction caused by abnormal nerve conduction due to ischemia-reperfusion (3–5).

### 3.3 The PNR process after PNI

Compared to the CNS, the peripheral nervous system possesses a degree of capacity for regeneration after injury (1). Waller degeneration usually occurs in the distal nerve after PNI. It mainly involves the degeneration and collapse of the local axon and myelin in the distal segment of the injured nerve, originating from the cell body, accompanied by various cytokines produced simultaneously (59).

The restoration of PNI typically entails three essential processes: eliminating incomplete myelin debris, generating new tissue, and recovering nerve impulse conduction function (13). The process of myelin debris removal commences with the degeneration and collapse of the impaired myelin sheath, prompting Schwann cells to phagocytose the myelin debris (60). Concurrently, these cells recruit a substantial quantity of inflammatory cells, including macrophages, neutrophils, and others, to expedite the clearance of the remnants (61, 62). Subsequently, ~28 days post-injury, T lymphocytes congregate at the distal end of the damaged nerve to elicit an immune response, thereby averting malignant inflammation at said location (63). Generating new tissue encompasses various events, such as the migration of neurons and elongation of axons, along with the proliferation and migration of Schwann cells around neurons. Additionally, it involves the regeneration and reconstruction of blood vessels and support systems surrounding nerve tissues (13, 64, 65). The restoration of nerve conduction function includes the proliferation of Schwann cells activated by cytokines and re-encloses nerve axons to form myelin sheaths. Ultimately, the ends of nerve axons establish correct connections with target organs and tissues, enabling proper functionality (66).

### 3.4 Peripheral nerve regeneration-associated genes

**Figure 1:**
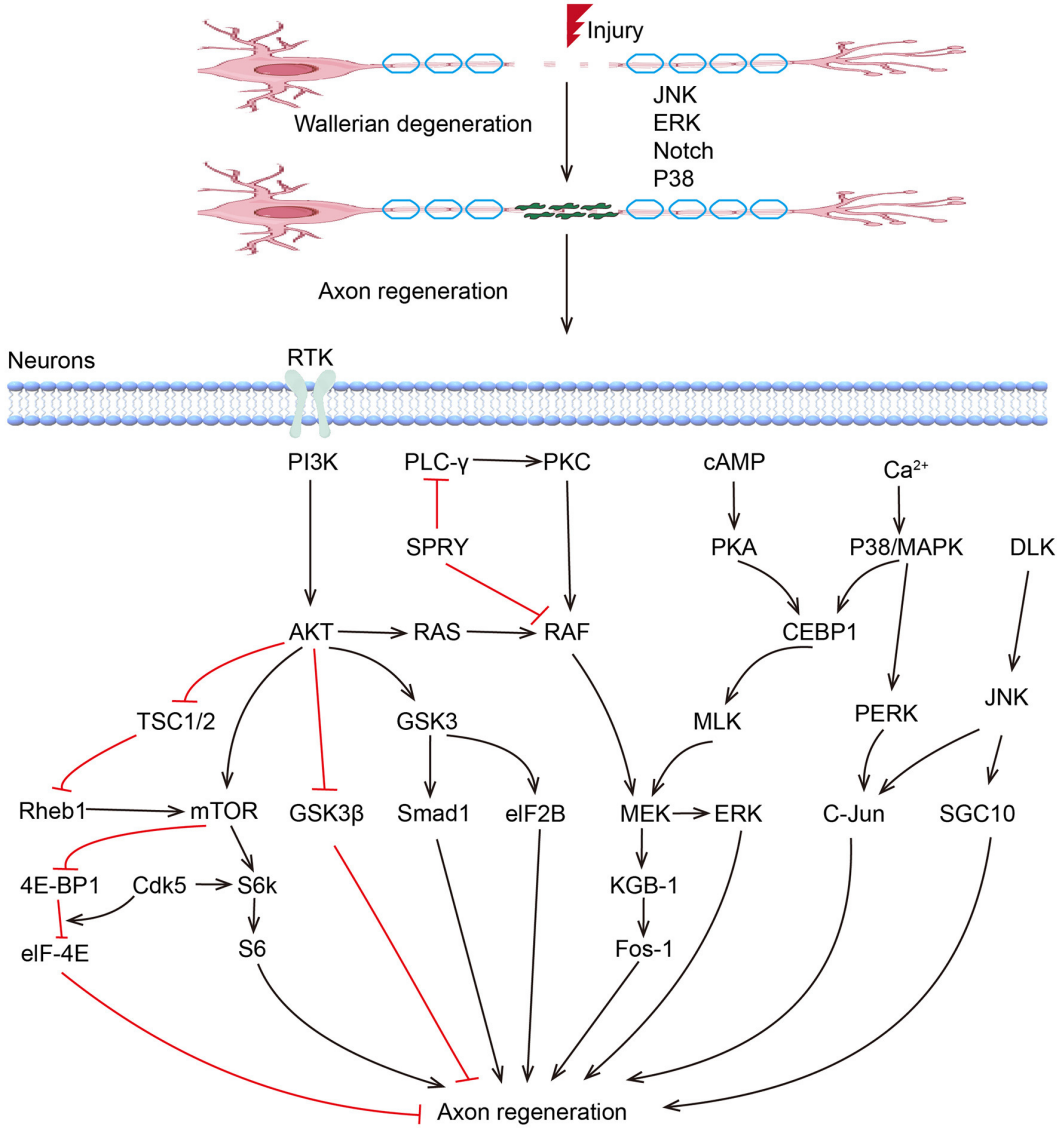
Kinase pathways regulate axon regeneration.

To date, an increasing number of Regeneration Associated Genes (RAGs) have been reported to play critical regulatory roles during peripheral nerve regeneration. These RAGs can be classified into several groups, including transcription factors, growth factors, miRNAs, and other non-coding RNAs (Figure 1).

#### 3.4.1 Transcription factors

Transcription factors are the main regulatory factors for various cellular processes under physiological and pathological conditions. Many transcription factors that are differentially expressed after peripheral nerve injury play essential roles in nerve regeneration, including c-Myc, Sox11, STAT3, Atf3, c-Jun, Smad1, Sox2, Krox20, Sox10, and p53 (67). Sox11 is a protein with an HMG domain that can effectively promote peripheral nerve regeneration. It regulates multiple genes, including adhesion molecules, cytoskeletal elements, growth factors, cytokines, neuropeptides, and other molecules related to regeneration (68). Bcl11a is crucial for Schwann cell activation and peripheral nerve regeneration. It participates in regulating Schwann cell activity via regulating the expression of Nr2f2. Reduction of Bcl11a in damaged peripheral nerves leads to restricted axonal extension and myelin sheath wrapping, reduced Schwann cell proliferation and migration rates, and impaired ability of debris clearance, thereby causing recovery failure (69).

#### 3.4.2 Growth factors

Growth factors (GFs) are neurotrophic factors known for regulating cellular proliferation, migration, and differentiation. In preclinical trials, exogenous application of GFs to the lesion site of peripheral nerves has demonstrated their high potential in repairing peripheral nerves by promoting myelin debris clearance, axonal sprouting, remyelination, neurogenesis, and neovascularization (70–72). These GFs, including nerve growth factor (NGF), brain-derived neurotrophic factor (BDNF), and neurotrophin 3 (NT-3), have been shown to stimulate axonal regeneration both *in vitro* and *in vivo* (73, 74).

#### 3.4.3 MicroRNAs

MicroRNAs (miRNAs) have been found to exhibit significant expression variation after peripheral nerve injury, reflecting their essential roles during this process. MiRNAs are prevalently involved in various aspects of peripheral nerve regeneration, encompassing inflammation, cell proliferation and migration, neurite outgrowth, and axon remyelination (75). MiRNA182 was found to be decreased in the initial stage of acute nerve injury, allowing for essential inflammatory reaction and increased SC migration by targeting fibroblast growth factor 9 (FGF9) and Neurotrimin (NTM) (76). Let-7 family can be upregulated after nerve lesion, resulting in the downregulation of Notch1 and higher levels of EGR2, consequently inducing remyelination (77, 78).

#### 3.4.4 Other non-coding RNAs

Apart from miRNAs, other non-coding RNAs, including LncRNAs and Circ-RNAs, are also involved in PNR regulation. Silc1 is a lncRNA that is highly expressed in neuronal tissue. Previous studies have shown that Silc1 regulates nerve regeneration by cis-activating Sox 11 (79). Knockdown of lncRNA Bc088327 inhibits cell viability of SCs and induces apoptosis and cell cycle arrest in S-phase. This lncRNA may also interact with Hereglin-1β, which is involved in nerve regeneration (80). Circ-Spidr is enriched in the cytoplasm of dorsal root ganglion (DRG) neurons. It can regulate the PI3K-AKT pathway and inhibit axonal regeneration of DRG after sciatic nerve injury (81). Overexpression of Circ-Ankib1 was reported to affect SC proliferation and axonal regeneration after sciatic nerve injury by directly binding to miR-423-5 p, miR-485-5 p, and miR-666-3p and regulating Cyp26b1 expression (82).

## 4 Kinase and PNR

The microenvironment of PNS undergoes alterations following nerve injury, including cellular damage and subsequent release of cellular constituents, leading to significant changes in the surrounding microenvironment of the injured tissue. Then, diverse cytokines and kinases are induced and participate in the clearance and regeneration process. Kinases, which regulate numerous signaling pathways and serve as crucial signaling molecules, are intricately associated with the regeneration of injured peripheral nerves, establishing the microenvironment necessary to regenerate injured nerve tissues (83, 84). It influences the repair pace for damaged peripheral nerves by modulating various processes, including autophagy, inflammatory response, cell apoptosis, cell cycle, and oxidative stress. Due to the involvement of multiple pathways, kinases have the potential to both facilitate and impede the regeneration process of injured peripheral nerves via regulating different biological processes.

### 4.1 Kinase affects the PNR by regulating the autophagy process

In the context of PNR, autophagy serves as a crucial regulatory factor. Studies have pointed out that the serine/threonine kinase, known as the mammalian target of rapamycin (mTOR), exerts significant regulatory influence on the autophagy process and neuronal protection (85). Suppression of mTOR activity in mice led to a substantial reduction in the phosphorylation level of downstream p70S6K protein and, concurrently, increased expression levels of autophagy genes *lc3* and *beclin1*, thereby facilitating autophagy and subsequently expediting the elimination of myelin debris and the regeneration process (86, 87). DLK1, a conserved MAPKKK, is an essential injury sensor regulating regeneration in motor and mechanosensory neurons by activating autophagy through a MAP kinase cascade (88).

### 4.2 Kinase affects PNR by regulating the inflammatory response

Inflammation is closely associated with the regeneration of injured nerve tissue and assumes a dual role, either positive or negative, during this process. It is widely acknowledged that the prompt pro-inflammatory reaction following the PNI is an indispensable prerequisite for eliminating tissue debris and facilitating effective regeneration (59). Kinases are closely associated with the inflammation response of the injured nerves. Both inflammation and nerve injury recovery can be controlled by p38 MAPK, which plays an essential physiological role in nerve regeneration. It was found that after sciatic nerve injury, the elevation in p38-MAPK phosphorylation levels inhibited the synthesis of IL-1β and TNF-α and positively influenced the regeneration of impaired tissues (89). The activation of IKK kinase facilitates the upregulation of downstream NF-κB pathway factors, attenuating the inflammatory response during PNI and fostering nerve regeneration (90, 91). Kinase DLK (Map3k12) assumes a pivotal role in the initial phase of nerve damage, exerting control over various downstream signaling molecules, including immune factors Csf1, Sarm1, and JNK/c-Jun, thereby governing the process of damage repair (92–95).

### 4.3 Kinase affects PNR by regulating cell apoptosis

Enhancing anti-apoptosis effects is advantageous for facilitating the restoration of impaired nerves. Rat c-Jun N-terminal kinases (JNK) and extracellular signal-regulated protein kinase (ERK) have been demonstrated to effectively inhibit the PI3K/AKT/mTOR signaling pathway, thereby reducing the expression of apoptosis-related proteins such as Bax and cleaved-Caspase3 (96, 97). Inhibited PI3K/AKT/mTOR signaling pathway was found to hinder scar tissue formation in the sciatic nerve after injury, thereby creating ample room for nerve regeneration (98). Additionally, inhibiting the activity of mTORC1 may reduce the BCL-2 expression and stimulate apoptosis, thereby negatively regulating the development of diabetic peripheral neuropathy (99).

### 4.4 Kinases affect PNR by regulating the cell cycle

Alteration of the cell cycle of nerve cells is also one of the most important factors influencing the regeneration process of injured nerves. In the central nervous system, AKT kinase can enhance the expression of cyclin D1 by deactivating glycogen synthase kinase-3β (GSK-3β) and reducing protein 27 kinase inhibitor 1 (p27kip1), thereby modulating the cell cycle, influencing cell proliferation rate, and impacting myelin sheath regeneration (100–102). Suppression of AKT phosphorylation can easily result in the arrest of the G1 phase, leading to cell apoptosis and hindering the subsequent recovery of the injured nerves (103). Peripheral nerve extrusion and severance resulted in a notable increase in the expression of protein kinase SKP2 while simultaneously degrading the downstream p27kip1 protein, which had an impact on the cell cycle of cells involved in the regeneration process, ultimately leading to alterations in the progression of injured nerve regeneration (104). The elimination of cyclin-dependent kinase CDK in peripheral nerves typically prevented Schwann cells from initiating the cell cycle, resulting in a significant decrease in the proliferation rate of Schwann cells. Consequently, the reduction of the proliferative Schwann cells severely impeded the regeneration of the damaged nerve myelin sheaths, which in turn affected the recovery of nerve function in the later stage (105).

### 4.5 Kinase affects PNR by regulating oxidative stress

PNI induces neurotoxicity, and the involvement of the oxidative defense system in nerve tissue can significantly enhance the efficacy against neurotoxicity. It was found that decreased JNK by silymarin (SLM) upregulated the expression of cyclic adenylate response element binding protein CREB, augmented the activity of superoxide dismutase and catalase, reinforced the antioxidant defense system, and suppressed apoptosis and inflammation, thereby safeguarding peripheral nerves (106). Another study has demonstrated that FGF21 might inhibit excessive ERK activation, thereby reducing cellular oxidative stress and autophagy and consequently improving remyelination and nerve regeneration after PNI (107). In addition, the alteration in PI3K kinase activity is intricately associated with excessive oxidation and apoptosis of Schwann cells (108–110).

### 4.6 Roles of hexokinase in PNR

High glucose has been recognized as one of the most influential factors causing diabetic peripheral neuropathy (DPN) with significant peripheral nerve damage (59). High glucose concentration can induce Schwann cells' apoptosis by affecting various cellular aspects, including oxidative stress, inflammatory reactions, endoplasmic reticulum stress, autophagy, nitrification, and signaling pathways (110). Hexokinase is the first enzyme that catalyzes the phosphorylation of glucose in glycolysis. It has been found that blockade of the enzymatic activity or disruption of the location of hexokinase significantly inhibited the neurite outgrowth of the sensory neurons (111).

## 5 Kinase affects PNR by regulating the function of different cells in PNS

Kinases are intricate enzymes, the mechanism of which is multilayered, complex, and cell-dependent. Here, we will briefly discuss the functional roles of kinases depending on the different cell types involved in PNR.

### 5.1 Kinase regulation of neurons

MAPKs play critical roles in peripheral nerve regeneration (Figure 1). Previous studies have demonstrated that P38 and JNK pathways are coordinated to regulate axon regeneration, which involves the participation of other kinases, including DLK1 and MLK1. MLK1/MEK-1/KGB-1 (JNK) MAPK Pathway is necessary for axon regeneration by facilitating the growth cone initiation and migration (112). ERK/MAPK and PI3K/AKT signal channels were found to be both activated in facial neurons after injury (113). The activation of ERK is necessary in proximal and distal nerve stumps, which promote neurite outgrowth and regeneration (114, 115). The Raf-ERK pathway was proved necessary for axon elongation in sensory neurons, while the PI3K-AKT pathway increases axon caliber and branching (116–118). Based on optogenetic systems, activated ERK and AKT successfully enhanced the axon regeneration in the peripheral nerve system in *Drosophila*, with an excellent ability to fine-tune and guide the axon regrowth, suggesting their application potential in treatments of peripheral nerve injuries (119). The role of GSK3 in PNR seems to be controversial (120). Although sustained GSK-3 without inhibition of PI3K/AKT was found to promote regeneration after sciatic nerve crush (121), blocking of GSK-3β enhanced axonal regeneration and remyelination in both CNS and PNS (122–124). In addition, Rho-kinase plays a negative role in neurite formation and maintenance in both CNS and PNS (125, 126). ROCK/ROCK pathway is involved in rearranging the cytoskeleton, and inhibition of the RhoA signal can counteract the inhibitory effects of other inhibitor molecules on axon growth, leading to increased axon sprouting and improved functional recovery (126, 127).

### 5.2 Kinase regulation of SCs

**Figure 2:**
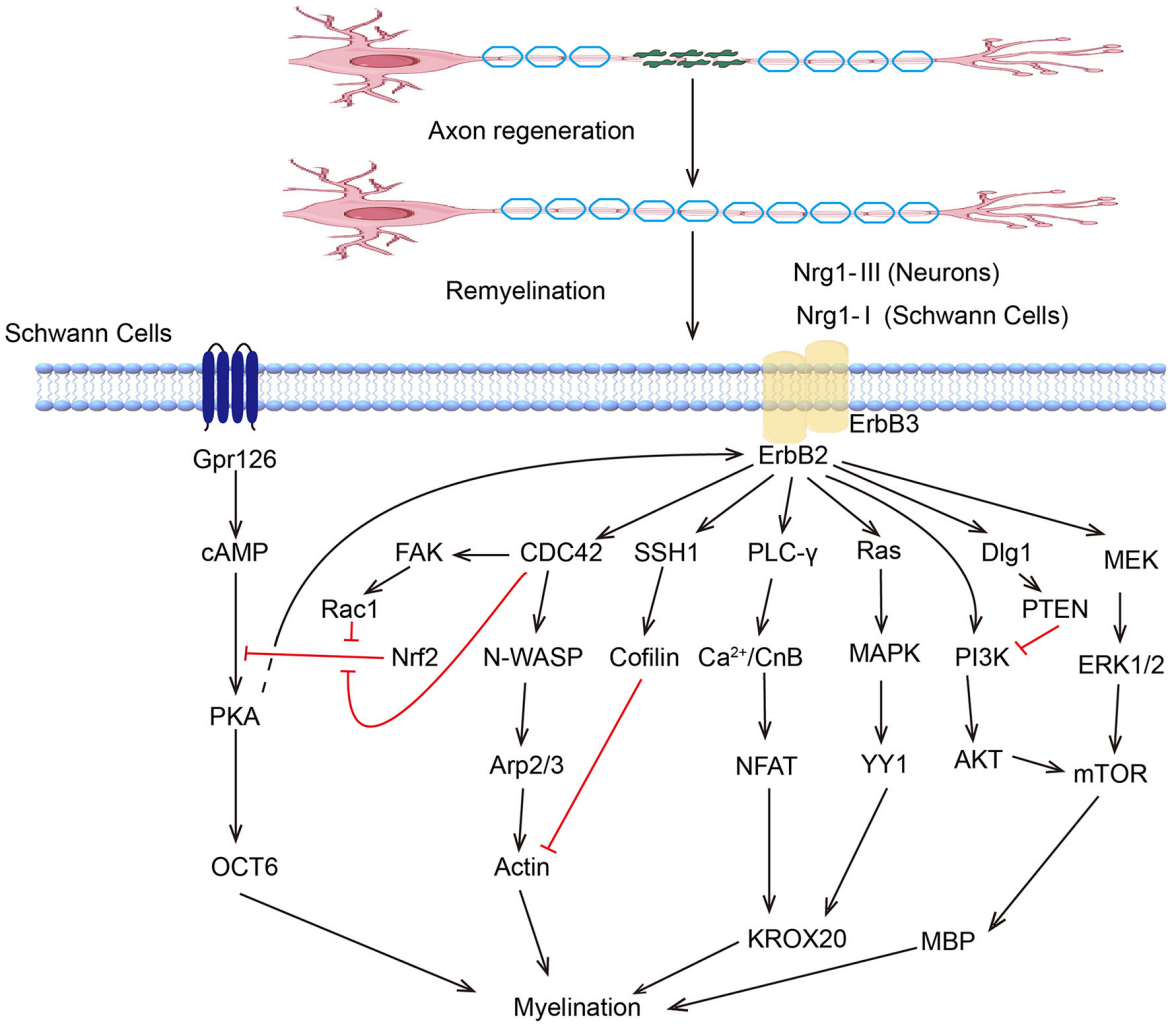
Kinase pathways regulate myelination of Schwann cells.

Peripheral nervous system SCs are renowned for their regenerative ability. Following the inflammatory response, the SCs proliferate rapidly and reinnervate the targeted area with new myelin sheaths, which involve various kinase signaling pathways (Figure 2). The ERK/MAPK pathway is critical in SCs, particularly following nerve injury. ERK activation leads to the upregulation of genes associated with myelin breakdown and nerve repair. This kinase is essential for the proliferation and migration of SCs and their dedifferentiation and redifferentiation processes, which are essential for PNR (128). Studies have shown that melatonin can induce dedifferentiation and proliferation of SCs through the Ras/Raf/ERK, MAPK, and GDNF/PKC pathways, suggesting its potential therapeutic value in treating PNI (129). The PI3K/AKT pathway can promote cell survival and proliferation, enhancing the regenerative potential of SCs. Stab1 was found to activate PI3K/AKT activity, inhibit SCs apoptosis, and promote PNR (130). Sam68 also participates in SC proliferation and regulates regeneration after sciatic nerve compression by activating PI3K/AKT activity (131). The AKT/mTOR pathway in neurons is crucial for myelin sheath growth by supporting protein translation and myelin protein synthesis (132). Mek/ERK1/2-MAPK and PI3K/AKT/mTOR signaling were found to affect Schwann cells' differentiation, myelination, and dysmyelination in either synergetic or independent ways (133). JNK, another member of the MAPK family, regulates the dedifferentiation and apoptosis of SCs and the expression of RAGs (134).

### 5.3 Kinase regulation of macrophages

In macrophages, P38 MAPK serves as a pivotal kinase in regulating inflammatory cytokine production while also facilitating the transition of macrophages from a pro-inflammatory M1 state to a pro-regenerative M2 state at the site of nerve injury through the modulation of AKT activity. This phenotypic shift is essential for mitigating inflammation and establishing a milieu conducive to nerve regeneration (135, 136).

### 5.4 Kinase regulation of fibroblasts

Various tissues throughout the body contain fibroblasts, which are mesenchymal cells that synthesize extracellular matrix (ECM) and basement membrane (BL) (137). The wound-healing process in injured peripheral nerves is highly regulated and involves fibroblasts. Transforming growth factor beta-activated kinase 1 (TAK1) can be activated by TGF- β, thereby affecting fibroblast activity and regulating the production of extracellular matrix and scar tissue formation (138).

### 5.5 Kinase regulation of endothelial cells

TEK receptor tyrosine kinase (Tie-2) controls endothelial-pericyte adhesion to maintain vascular integrity. Tie2 kinases in endothelial cells play critical roles in angiogenesis during nerve regeneration. They facilitate the response to growth factors, such as VEGF, to stimulate the formation of new blood vessels, ensuring the delivery of essential nutrients and oxygen to regenerating nerves (139, 140).

## 6 Conclusion

PNI diseases are frequently encountered as accidental conditions. Despite the relatively well-understood mechanisms and enhanced precision in surgical interventions, a practical clinical treatment approach still needs to be discovered, leading to suboptimal functional recovery in the long term. PNI frequently induces alterations in kinase activity and tissue inflammation, influencing the subsequent reparative processes. However, the regeneration of peripheral nerve damage is influenced by numerous factors, including the extent of nerve severance, the length and depth of the nerve defect, the level of wound inflammation, the patient's age and physical condition, as well as the activity and expression of various kinases, transcription factor expression and regulation, and nutrient factor secretion. Considering the complexity of these factors, it is evident that more than one factor alone is needed to comprehensively and effectively address the problem. Hence, to overcome the challenges associated with the disease, the examination of kinase function and effectiveness within the framework of nerve injury restoration, alongside the simultaneous analysis of diverse contributing factors, will present a potential avenue for providing innovative perspectives and strategies in managing PNI conditions. In the future, the efficacy of clinical treatment could also be enhanced by identifying drugs that target the kinase enzyme activity.

## Author contributions

XZ: Funding acquisition, Writing—original draft. XD: Writing—review & editing. XL: Conceptualization, Writing—review & editing.
